# Barriers towards Sun Exposure and Strategies to Overcome These Barriers in Female Indoor Workers with Insufficient Vitamin D: A Qualitative Approach

**DOI:** 10.3390/nu12102994

**Published:** 2020-09-30

**Authors:** Nurul Nadiah Shahudin, Mohd Jamil Sameeha, Arimi Fitri Mat Ludin, Zahara Abdul Manaf, Kok-Yong Chin, Nor Aini Jamil

**Affiliations:** 1 Faculty of Sports Sciences & Recreation, Universiti Teknologi MARA (UiTM) Cawangan Pahang (Kampus Jengka), Pahang 26400, Malaysia; p97542@siswa.ukm.edu.my; 2 Centre for Community Health Studies (ReaCH), Faculty of Health Sciences, Universiti Kebangsaan Malaysia, Kuala Lumpur 50300, Malaysia; sameeha@ukm.edu.my; 3 Centre for Healthy Ageing and Wellness (H-CARE), Faculty of Health Sciences, Universiti Kebangsaan Malaysia, Kuala Lumpur 50300, Malaysia; arimifitri@ukm.edu.my (A.F.M.L.); zaharamanaf@ukm.edu.my (Z.A.M.); 4 Department of Pharmacology, Faculty of Medicine, Universiti Kebangsaan Malaysia, Cheras 56000, Malaysia; gabrielchinky@gmail.com

**Keywords:** vitamin D, sunlight exposure, barriers, indoor workers, female, focus group discussion

## Abstract

The prevalence of vitamin D insufficiency is significant even in tropical countries such as Malaysia. Sun exposure is the primary source of vitamin D for most people due to limited intakes of food containing vitamin D and supplements. This study explored the perception of barriers towards sun exposure and strategies to overcome these barriers among vitamin D insufficient women workers in Kuala Lumpur, Malaysia. Twenty-five female indoor workers with serum 25-hydroxyvitamin D < 50 nmol/L participated in seven focus group discussions (FGDs). Barriers towards sun exposure were lack of accurate knowledge of vitamin D, health concern towards sun exposure, time constraints, desire to have fair and beautiful skin, sedentary lifestyle, indoor workplace, weather, lack of social support, living arrangement, safety concerns, and religious or cultural practices. The improvement strategies were classified into lifestyle changes and workplace opportunity for sun exposure. Public education on safe sun exposure to produce an optimal level of vitamin D is necessary. Future studies should evaluate the effectiveness of sunlight exposure program at workplace for the high-risk vitamin D deficiency group.

## 1. Introduction

Vitamin D insufficiency remains to be a concerning health issue worldwide due to its high prevalence among populations from various countries, cultural backgrounds and age groups [1], including tropical countries receiving substantial amount of sun exposure throughout the year [2]. However, the significant health impact of low vitamin D status remains unclear. Although incidence of rickets and osteomalacia are increasing in certain places, these conditions remain relatively rare worldwide [3,4]. The clinical evidence of vitamin D deficiency and nonskeletal health is not yet validated [5]. In countries where dietary vitamin D food intake and supplements are limited [6], and fortification of food with vitamin D is not compulsory, sunlight exposure is the primary source of vitamin D among the populations [6,7]. Government bodies often establish sun exposure guidelines to ensure a balance between the beneficial and harmful effects of sunlight [7]. However, it is almost impossible to provide guidance that fulfils all aspects because several factors can influence the cutaneous synthesis of vitamin D such as the zenith angle and latitude, different times of the day, air pollution, skin pigmentation, body surface area exposed when outdoors and age [8,9]. Currently, there are no sun exposure guidelines in Malaysia [10].

Previous studies around the world suggest that vitamin D deficiency is attributed to limited sun exposure, lack of dietary vitamin D food intake [11,12], urbanization [12], air pollution [13], obesity [14] and sedentary lifestyle [15]. It has been speculated that the excess body fat retains vitamin D in the body fat compartments resulting in decreased bioavailability of vitamin D among the obese individuals. This could also explain the lower vitamin D status among females compared to males as a result of higher body fat mass in females [16]. The limited sun exposure, especially in tropical countries, such as Malaysia, Thailand, Saudi Arabia and Iran, is mainly due to sun avoidance practice that is influenced by cultural, racial and religious backgrounds [2,6,8,9]. Malaysia is a unique multi-racial country, which is mainly composed of Malays, Chinese and Indians with various skin types. The high-risk groups of vitamin D insufficiency in Malaysia are children [17], adolescents [18,19], females [19,20,21], urban population [17,21], indoor workers [6,21], obese [17,18] and Indian and Malay ethnicities due to higher skin pigmentation and clothing styles (especially among Malay women wearing full-body garments) [2,6,17,19]. 

Cutaneous synthesis of vitamin D through outdoor activities and sun-bathing is widely practice by the westerners [9,11,22]. However, this practice was not popular among the vast majority of the Asian populations [23,24,25]. Sun-bathing and outdoor activities for sports and recreation are uncommon and not part of Asian culture [26,27]. Incidental sun exposure might occur while commuting to and from destinations, especially amongst those using public transportation. However, due to urbanization, most Asians are passive commuters and indoor workers [23,27].

A recent study on knowledge, attitude and practice conducted among high-risk female office workers in Kuala Lumpur, Malaysia suggested that further investigation into the factors contributing to vitamin D deficiency is essential as they have a moderate attitude and practice towards sunlight exposure and dietary intake of vitamin D [10]. This information that could be garnered from such studies would provide practical recommendations to the public and health agencies to prevent vitamin D deficiency among the Malaysian population. The current study is an extension of the previous study [10], which aimed to explore the perceived barriers towards sun exposure and strategies to overcome these barriers among indoor women workers with insufficient vitamin D status in Kuala Lumpur, Malaysia.

## 2. Materials and Methods 

### 2.1. Study Participants

The participants from this study were derived from a recent study conducted among women office workers working in a medical university and teaching hospital in Kuala Lumpur, Malaysia [10]. In brief, they were indoor women workers with insufficient vitamin D level (serum 25-hydroxyvitamin D (25OHD) concentration <50 nmol/L) [28] and aged between 18 and 59 years. Indoor workers are defined as those working in an indoor setting for at least four days a week. Purposive sampling technique was used to ensure participants were recruited from four groups: (i) administrative staff (clerical staff, administrative staff assistants and lab technicians); (ii) executive (officers and top management employees); (iii) academicians (lecturers, senior lecturers and professors); and (iv) clinicians (medical assistants, nurses and doctors). Pregnant, lactating, or menopause women were excluded from this study. This study was approved by the Research Ethics Committees of Universiti Kebangsaan Malaysia (approval code: UKM PPI/111/8/JEP-2019-116).

### 2.2. Focus Group Discussion (FGD)

The FGDs were conducted in Bahasa Malaysia between October and November 2019 in a meeting room. All participants answered basic questionnaires and had given their written informed consent prior to participating in the FGD. Each FGD was led by a trained moderator (NNS), monitored by an experienced qualitative researcher (MJS) and observed by a research assistant. The moderator audiotaped all sessions using a video recorder (Sony, ICD-UX560F, Japan), while the research assistant was tasked with taking notes and recording descriptions of the participants’ non-verbal behaviors. These notes offered as a backup to resolve any issues regarding audio clarity and to monitor participants’ body language during the session.

The moderator first explained the purpose of the discussion, rules and regulations of FGD, followed by asking a series of specific, predetermined questions (Table 1). Two main topics were discussed, namely (i) barriers to receiving adequate sun exposure to synthesise vitamin D; and (ii) strategies to improve their vitamin D insufficiency. All participants were encouraged to share their ideas and opinions, and suitable probes were used to obtain in-depth findings. Recruitment was stopped when no new relevant information emerged due to data saturation. 

### 2.3. Data Coding and Analysis

The Consolidated Criteria for Reporting Qualitative Research (COREQ) framework [29] was used to guide the reporting of the findings. Audio recordings were transcribed verbatim. The transcripts were thematically analyzed using NVivo version 12 [30]. Each transcript was reviewed line-by-line and codes were categorized concurrently by three coders (NNS, NAJ and MJS). The identified codes were either single words (e.g., hot, beauty) or short phrases (e.g., sunscreen usage) that captured the essence of the excerpts [31]. Subsequently, the codes were grouped under broad domains of the discussion guide and theoretical constructs (e.g., time constraints). The discrepancies in coding were discussed with the research team (NNS, NAJ, MJS, AFML and ZAM) until a mutual agreement was achieved for the final nodes as described by Hadi and Closs [32]. As all researchers had expertise in nutrition and exercise health, they attempted to suspend their perspectives to avoid biases and focused on participants’ statements that described their perceptions and experiences during the FGD.

### 2.4. Trustworthiness

To ensure the quality and trustworthiness of this study, multiple approaches were used. Prolonged engagement with study participants helped to gain their trust and establish rapport [32], as the researcher worked with the same participants during the preliminary study in February to May 2019 [10], until the FGD session conducted in October 2019. An audit trail was used to improve the quality of the instrument [32]. The lead researcher (NNS) met with supervisors (NAJ & MJS) after the first two FGDs to discuss possible questions to be revised and checked on probes improvement. Furthermore, peer debriefing was applied where the weekly meetings were conducted between NAJ, MJS and NNS to discuss on data analysis and interpretations continuously throughout the research process. Lastly, thick description of this study was presented in the methodology section to obtain external validity to ensure that this study could be transferable to other settings, situations and populations [32].

## 3. Results

Twenty-five female indoor workers took part in seven FGDs. Each group contained three to five participants and ranged from 45 min to 1 h in duration. Table 2 shows the participants’ characteristics. Majority of the participants were between 30 and 39 years old (64%), married (64%), had a diploma or a higher education background (80%) and worked as an administrative staff (76%). Most of them had low to middle household-income (92%).

### 3.1. Barriers towards Sun Exposure

A total of eleven perceived barriers towards sun exposure for adequate vitamin D status were identified and categorized into internal and external factors (Table 3). Five themes were recognized from the internal factor, including lack of knowledge due to misinformation about how vitamin D is synthesized upon exposure to sunlight or ultraviolet B (UVB) irradiation. Health concern was another barrier towards sun exposure, which was further classified into pre-existing medical conditions and risks for skin cancer. Other internal barriers towards sun exposure were time constraints associated with family and work commitments, desires to have fair and beautiful skin and sedentary lifestyles. 

For the external factor, six themes emerged including indoor workplaces, hot weather and unpredictable climate change. Lack of social support from the spouse, family members or friends to do outdoor activity together reduced the participants’ interest to be exposed to the sun. Limited direct access to the sun at home as a result of living arrangement, house design and location of the house situated in the city were also mentioned. Finally, safety concerns and cultural and religious practices among Muslim women were also barriers towards sunlight exposure. 

### 3.2. Strategy to Overcome the Barrier towards Sun Exposure

**Figure 1:**
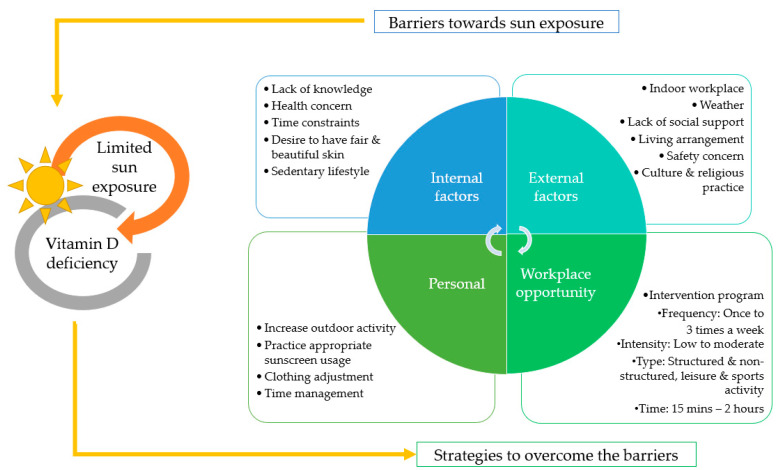
Barriers and improvement strategies to improve sun exposure for vitamin D.

The discussion on the strategies to overcome the barrier towards sun exposure was classified into two factors (Table 4). The first is focusing on the personal improvement strategies that the participants were willing to perform on their own by changing their sun exposure behaviors to improve their vitamin D status. Suggestions included increasing outdoor activities during the weekends, practicing appropriate sunscreen usage, clothing adjustments that increase body surface area (BSA) exposed to sunlight, and improving their time management. The second part of the discussion is followed by suggestions for a suitable intervention program to be conducted by employers. The recommendations were categorized based on themes which include types, frequency, intensity and time that can be done based on workplace settings. Figure 1 summarizes the factors of barriers towards sun exposure and improvement strategies as identified by the indoor female workers in this study.

## 4. Discussion

This study used a qualitative method of FGD to explore the barriers and improvement strategies towards sun exposure among women working indoors in the capital city of Malaysia with insufficient vitamin D status. The study provides new knowledge in this area, with practical messages that can be applied to the population at risk of vitamin D deficiency with a similar demographic background.

Lack of knowledge is on the top of the list for barriers towards sun exposure. We found that most of the participants had a misconception on the metabolism of vitamin D including how vitamin D is synthesized, and the differences between ultraviolet A (UVA) and UVB radiation. UVB is the sources of cutaneous synthesis of vitamin D and it cannot penetrate glass [11]. The participants assumed that the UVA they are exposed to while driving in the car and sitting by office window (with glasses) might provide them with vitamin D. A previous study done in a Malaysian sub-urban setting among post-menopausal Malay women found that poor knowledge on vitamin D influenced their sun exposure behavior [33]. The similarity of the findings could be owing to the lack of knowledge regarding vitamin D health benefits compared to other groups of vitamins among both urban and sub-urban females in Malaysia [1,6,33].

Health concerns to sun exposure, particularly increased risk of skin cancer, were mentioned in the FGD. According to the Global Cancer Observatory 2018 report, skin cancer was the 30th most common cancer in Malaysia and is not as prevalent as other cancers [34]. Most participants believed that they are susceptible to skin cancer due to the thinning of the ozone layer. They were not aware that skin cancer is not prevalent among Malaysians, especially the Malays. Our participants practiced poor sun protection behaviors, such as inappropriate amount and timing of sunblock application. They usually apply sunblock once in the morning together with their makeup before going to work. This finding is consistent with a previous study of skin cancer prevention practice among 400 university students in Kuala Lumpur, with the majority of the subjects being Malay women [35]. The study found that only 43.5% of the participants used sunblock, often applying them inadequately and forgetting to reapply after swimming, sweating, or other activities after the sunblock is degraded or washes-off [35]. A cross-sectional study in Queensland, Australia where the highest rate of skin cancer in the world reported that participants from low-income household tend to have uncertainty and concern about vitamin D and sun exposure [36]. Both studies suggested that sun protection education is needed in these populations to address the misunderstanding about skin cancer risk and improve the skin protection practice [25,35]. Some participants in our study also perceived that dizziness and fever were direct results of sun exposure, despite the lack of scientific evidence. This assumption also led to sunlight avoidance among our study participants.

Time constraint was frequently mentioned by the participants that caused limited sun exposure. All of the subjects in this study were working women in the urban area, who thrive to balance between work and family. This finding is consistent with a recent study among employees in Kuala Lumpur, who associated ‘lack of time’ with being busy with work, house chores and other family commitments [37]. A recent review highlighted that limited time spent outdoors among the urban population was due to their working nature, increased screen time and less manual work undertaken outdoor compared to rural population [12]. The majority of our participants drove to work. A typical driving scenario in the urban area encompasses a mixture of standstill traffic and slow traffic, depending on the route and time of the day [38]. Our participants spent on average around 1 to 1.5 h to commute daily to work. Once they reached home, they were bound to do house chores, further limiting their sunlight exposure time. Similar experiences were shared by women in eight European countries, whereby gender inequality and expectations of married working women, especially mothers, are higher in balancing work-family commitments compared to their counterparts [39].

The subjects of this study also expressed aesthetic concerns about sunlight exposure. This finding echoed previous reports among Asians, whereby a fair skin tone is often associated with beauty [25,40]. Common remarks such as fear of becoming dark, having freckles, sunburn and makeup usage, influenced sun avoidance behavior, especially among women [25]. Apart from sun protection cream, most moisturizers, foundation cream and compact powder these days, come with an added sun protection factor (SPF). The subjects surveyed also indicated a preference to stay indoors. A sedentary lifestyle has long been associated with vitamin D deficiency, physical inactivity, and health-related problems such as obesity and diabetes [2,19,20]. While the mechanism underlying the association between vitamin D and obesity is still uncertain, the low vitamin D status in obese individuals could be due to their sedentary lifestyle and low outdoor activity, vitamin D sequestration in adipose tissue, or simply a volumetric dilution effect [14]. A population study in Malaysia reported that only 14% of adults in Malaysia ever exercised and the majority spent 74% of the day being sedentary such as watching television, lying down, or hanging out to have drinks [41]. These factors are also correlated with our participants’ social support given by their spouse, friends and employers [42]. It was previously reported that single individuals preferred to stay at home when there was no company while married couples with kids mainly adhered to their family commitments [37].

Being bounded indoors at their workplace as well as living arrangements are among the external factors leading to a lack of sunlight exposure. On average, indoor workers spend eight hours a day at the office for five days a week during the day. A similar investigation in Singapore found that indoor workers were among the high-risk group for vitamin D deficiency, probably because UVB is filtered by the glass window of the office [43]. Similar to other densely populated cities like Hong Kong and Singapore, the majority of the populations in Kuala Lumpur live in high-rise buildings [44], due to higher land costs. Low-income groups typically reside in a flat unit or a low-cost apartment with basic facilities, whereas the middle- and high-income groups may opt to live in a condominium with full facilities such as a playground, in-house park for jogging or walking, tennis court and swimming pool. The majority of our participants who lived in high-rise buildings mentioned that they either did not receive direct sunlight from their unit, or did not have sports or recreational facilities from their surrounding vicinity to encourage them to go out. A study among Saudi women attributed the lack of sun exposure to the modern house designs. Currently, the house design in Saudi has changed towards closed and high rise buildings built without a balcony, thus, limiting their sun exposure compared to living in a home with older designs that often incorporate a courtyard that allows sun ray to enter the house [45]. Apart from geographical reasons, the external barrier was influenced by cultural and religious practices similar to those of Muslims in our study. The Malays in Malaysia are generally Muslims. As a Muslim woman, specific clothing guidelines commonly observed based on Quranic teachings allows only the face and hands to be exposed when in public [45,46]. Our participants further highlighted that religious restrictions that affect their actions and activities in public also limit their sun exposure.

Outdoor safety is an emerging barrier towards sun exposure among our subjects. The rising cost of living in Kuala Lumpur has given rise to an increased crime rate in the vicinity. Based on the criminal index in Malaysia from the year 2009 to 2015, 16,034 street crime investigations were carried on snatch thefts, robberies and similar offences [47]. These crimes were almost exclusively targeted on women walking alone in open areas [47]. Our participants mentioned that they too felt worried and insecure about being outdoors, even in public parks.

Strategies to tackle the barriers to sun exposure were mentioned by the participants through two factors: personal improvement and workplace opportunity. First and foremost, most of the participants agreed that they need to change their lifestyle to improve their vitamin D status. An educational program is essential for the promotion and enhancement of personal improvements to sunlight exposure. Besides, participants also believed that employers could play an essential role in promoting sun exposure at the workplace. Activities such as Zumba, aerobics and light exercise may be suitable to be conducted at workplace to promote both sunlight exposure and physical activity. Various timing and frequency proposed by the participants, ranging from 15 min to 2 h, once to three times a week. Besides, employers could also provide outdoor workstations that allow for sun exposure while being at work whenever necessary. These suggestions, however, would be subject to approval by the employer and improvements in existing organizational policies. A structured program of gradual increment from low to high intensity activities for 30–60 min was suggested in previous study among desk-based employees in Kuala Lumpur, Malaysia [37]. However, dietary vitamin D intake and supplements may be recommended to make up the shortfall from sunlight exposure, especially among those who have limited sunlight exposure, for whatever reasons they may have. A recent data showed a positive association between adherence to the Mediterranean diet and vitamin D status that could be explained by the synergistic anti-inflammatory and antioxidant effects of its high consumption of whole grain and plant-based food and moderate intake of fish, white meat, and eggs [48].

This study provides insight into the barriers towards sun exposure among vitamin D insufficiency women. However, several limitations were noted in this study. Firstly, it was conducted in one institution, with an uneven representatives from four target groups; administrators, executives, academicians and clinicians, due to time and work commitment constraints. Therefore, the findings in this study mainly reflect the perceptions of the administrator group. This study was also conducted among the Malay women population only, thus, it does not reflect the perception of other ethnic groups in Malaysia. Future studies should explore the barriers towards sun exposure among other at-risk groups, such as shift workers in Malaysia to understand their perspective on this matter. Furthermore, an intervention study should be performed to assess the effectiveness of sunlight exposure program in the workplace in improving vitamin D status of female indoor workers in Malaysia.

## 5. Conclusions

The Malay female indoor workers with insufficient vitamin D level reported that the barriers to sun exposure were influenced by both internal and external factors, such as work commitments, environment, and social factors. An educational program should be mooted in spreading accurate information on the importance of sun exposure and the best practice for optimal level of vitamin D. Working women should also take the initiative to maximize their sun exposure during the weekends. Apart from lifestyle changes, employers also can play an active role in promoting positive sun exposure at the workplace by organizing outdoor activities. Regardless, further evaluation on the effectiveness of sunlight exposure program among female indoor workers remains necessary.

## Figures and Tables

**Table 1:** Focus Group Discussion Questions.

Topic	Questions
Barriers	(1) Internally, what do you think leads to your vitamin D insufficiency? (2) Externally, what do you think contributes to your vitamin D insufficiency? (3) What prevents or restricts you from sun exposure on weekdays? (4) What prevents or constraints you from sun exposure on weekends?
Improvement Strategy	(1) Do you want to improve your vitamin D status? (2) What are you going to do to improve your vitamin D level? (3) What type of activities do you want your employer to do to promote workplace sun exposure? (4) Can you suggest a suitable workplace intervention program? (frequency, intensity, type and time)

**Table 2:** Participants’ Characteristics.

Variable	*n*	(%)	Mean	SD
Age			35.6	5.8
18–29 years	3	12%		
30–39 years	16	64%		
40–49 years	6	24%		
Marital Status				
Single	6	24%		
Married	16	64%		
Divorced/widowed	3	12%		
Education				
Secondary school	5	20%		
Diploma	10	40%		
Bachelor or higher	10	40%		
Occupation				
Administrative staff	19	76%		
Executive	1	4%		
Academician	3	12%		
Clinician	2	8%		
Household Monthly Income (MYR *)				
Low (<3860)	12	48%		
Middle (3860–8319)	11	44%		
High (>8319)	2	8%		

* MYR: Malaysia Ringgit.

**Table 3:** Themes for barriers towards sunlight exposure.

Factor	Themes	Quotes
Internal factor	Lack of knowledge	“I got my vitamin D synthesized when I was driving. The sun rays were felt through the windscreen of my car,” (33 years old, clinician). “Vitamin D sun is the morning sun. The afternoon and evening sun do not help to produce vitamin D, right?” (27 years old, administrative staff).
	Health concern	“If we are exposed under the sun, will it cause skin cancer? That is why I prefer to stay indoors” (49 years old, administrative staff). “I have this stigma that sunlight can make me fall sick, get fever or headache, regardless whether it is in the morning, afternoon or evening’s sunlight,” (41 years old, administrative staff). “I can’t stand with the hot sun these days. It is so much different compared to younger days, and the heat was unbearable. I believe the sun rays these days is harmful and can cause cancer. It is because of the thinning ozone,” (32 years old, administrative staff). “I am a patient of cardiovascular disease. I had a minor stroke two years ago. That is why I didn’t go out much these days. Whenever I walk, I tend to get tired quickly, thus making me feel lazy (to walk),” (44 years old, administrative staff). “I am very much an indoor person because my health doesn’t permit (me to exercise). I must be cautious whenever (I’m) outside because my respiratory system is very sensitive. For example, if I were to go out at the park, it needs to be somewhere dust-free and less polluted because I can get sick quickly and (it) may cause prolonged cough for months,” (34 years old, executive).
	Time constraints	“I don’t have enough time to go out after work. There are times when I would only reach home at 7.00 p.m. due to heavy traffic,” (34 years old, administrative staff). I don’t have time to do any activities before or after work because I stay far from the office, approximately 30 km away. I usually reach home in an hour. Sometimes, I have to travel for 2 h,” (32 years old, administrative staff). “No time to go out on weekdays. I must fetch the kids after work. Once I reach home, I need to cook and do all the house chores. As for the weekends, I will do major cleaning, cooking and spend some family time at home. Plus, my husband works on the weekends. Most of the time, I just let the kids play at the car porch (covered) instead of going out,” (35 years old, administrative staff). “I don’t have time to do any outdoor activities on the weekends because I have part-time work commitment as a phlebotomist,” (33 years old, administrative staff).
	Desire to have fair and beautiful skin	“I wear long sleeves whenever I’m out because it’s hot and I’m afraid of becoming dark,” (37 years old, clinician). “I used to have a lot of freckles. My beautician said the UV rays could cause freckles. That is why I avoid sun exposure,” (35 years old, administrative staff). “I think because of ageing, I have been consistently using SPF moisturizer for the past three years to keep hydrated. So, I can avoid wrinkles and dry skin,” (42 years old, administrative staff).
	Sedentary lifestyle	“I don’t do any outdoor activities. The most I would do on the weekends is to hang out at eateries,” (33 years old, administrative staff). “I often spend time at home on the weekend watching television,” (24 years old, administrative staff). “I don’t know why, but even when I had the chance (to go outdoor), I prefer to stay at home or indoor activity,” (42 years old, administrative staff).
External factor	Indoor workplace	“I am confined indoors during work from 8.00 a.m. to 5.00 p.m. So, sun exposure is limited during the weekdays. I normally take my lunch in the office or cafe, which is in the same building. So, there is no reason for me to go out,” (41 years old, administrative staff). “I work in a diagnostic lab that is in the basement. I don’t see the sun during working hours,” (32 years old, clinician).
	Weather	“Malaysia’s weather is so hot. So, I prefer to stay indoors,” (33 years old, clinician). “Our weather is so unpredictable, especially during the rainy season. I skip being outdoors and choose to spend the time at my favorite eateries instead,” (37 years old, administrative staff).
	Lack of social support	“My husband works during the weekends. Normally, I would wait for his off days to go for an outing. Otherwise, I just spend time with my son at home,” (35 years old, administrative staff). “I am single and don’t have a lot of friends. I will only go out to the park if my nephews or nieces come over to visit me. I wish to go out more often, but I don’t have anyone to go with,” (37 years old, academician). “My husband is not an outdoor person. He loves to go shopping and eat at the mall. So, I have no choice but to follow him as we normally go there at least twice a week,” (32 years old, executive).
	Living arrangement	“I stay in a high-rise apartment, and there is no direct sunlight coming in. I don’t get sunlight because my apartment doesn’t come with a balcony. I even have to dry my clothes inside,” (32 years old, administrative staff). “I stay at an apartment on the fourth floor, and I don’t go out often even during the weekends, just too lazy to go down,” (31 years old, clinician). “I stayed in a sub-urban landed property. I don’t get direct sunlight in my house compound. There are a lot of big trees surrounding my house,”(44 years old, administrative staff).
	Safety concern	“I prefer indoor activities instead of outdoors because of safety reasons. For example, if you were to go out during the early mornings at the park, it tends to be too quiet. I am scared to go there alone,” (38 years old, administrative staff).
	Culture and religious practices	“I enjoy outdoor activities, but I don’t like it when men are staring at me (in public),” (32 years old, executive). “I am particular in covering my aurah (part of the body that is prohibited from being revealed to other men for Muslim women). I was active before, doing workout and even join an aerobic class. However, I will make sure that I follow the rules (only exposing the face and both hands up to the wrists),” (42 years old, administrative staff).

**Table 4:** Themes for strategies to overcome the barrier.

Factor	Themes	Quotes
Personal Improvement	Increase outdoor activity	“I am unsure whether I can do it on weekdays, but I will do more outdoor activities on the weekends. Maybe later in the afternoons after I am done with household chores,” (35 years old, executive). “I need to make some changes, for example, do outdoor activities,” (42 years old, administrative staff). “Outdoor outing with the family, for example a trip to the National Zoo. Not only we get to expose under the sun, but also (we) can spend quality time with the family,”(31 years old, administrative staff).
	Practice appropriate sunscreen usage	“The least I can do is to reduce my sunscreen usage, not to apply too thick, especially in the mornings,” (27 years old, administrative staff).
	Clothing adjustment	“I think I need to wear short-sleeved clothing instead of long-sleeved when I do my exercise so that I absorb more (sunlight),” (37 years old, clinician). “Just open (rolled up her sleeves) and expose it,” (49 years old, administrative staff).
	Time management	“I will do house chores at night. So, I can go out during the day,” (32 years old, administrative staff).
Intervention Program at Workplace	Type of activity	“Walking around the campus. For example, aim for the daily 10,000 steps,” (37 years old, academician). “Maybe just stay out, like sun-bathing with clothes on,” (32 years old, administrative staff). “Group activity such as treasure hunt around the campus,” (32 years old, administrative staff). “I guess an outdoor aerobic such as Zumba is the most suitable activity and we have done it before,” (33 years old, clinician). “I think leisure activities like ‘tele-match’ and family day is suitable,” (42 years old, administrative staff). “Maybe organize a run (paused) like the fun run. If it is conducted on weekends, we can include our family members too,” (33 years old, administrative staff). “If we take a look at the current surrounding, I think outdoor cleaning activity is suitable as one of the methods to obtain sunlight,” (33 years old, administrative staff). “Maybe the employer can provide an outdoor workstation that allows the staff to bring their laptop and do their work outdoors. Apart from the lab work which needs to be done indoors, I can do report writing and administrative related work outdoors,” (41 years old, administrative staff).
	Frequency	“I think once a week is fine. If it’s more than that, two or three times a week, that’s a bit too frequent. It will disrupt my work, and I believe my employer will not approve it,” (37 years old, administrative staff). “Twice a week is sufficient,” (24 years old, administrative staff). “I think once a week is not sufficient, maybe around two to three times a week,” (32 years old, administrative staff).
	Intensity	“I prefer to do light exercise. I don’t want to be drenched in sweat in the morning and I need to do work after that,” (37 years old, academician). “The exercise intensity should be moderate. So, we can maintain our fitness apart from getting sun exposure. I think it can be boring if it’s too slow and relaxed,” (34 years old, administrative staff). “I prefer low intensity, more relax,” (32 years old, administrative staff). “I prefer moderate (intensity) and gradually increase the intensity. Not only we get the benefit of sun exposure, but also at the same time (we) can improve our cardiovascular fitness,” (38 years old, administrative staff).
	Duration and time of the day	“I think the best time to do the program is between 8.00 a.m. to 9.00 a.m. before we start work. It is inconvenient to work, stop for exercise, then work again,” (42 years old, administrative staff). “Evening session is suitable, about 5.30 p.m. It is more relaxing to do exercise once the work is done,” (33 years old, administrative staff). “10.30 a.m. onwards is suitable as normally we are less occupied around that time,” (32 years old, administrative staff). “The first two hours between 8.00 a.m.–10.00 a.m. is fine to me,” (33 years old, administrative staff). “I think I can spare 15 min for that (intervention),” (24 years old, administrative staff). “Half an hour is workable for me,” (34 years old, administrative staff). “30 min is not enough, at least one hour per session,” (34 years old, administrative staff).
